# Mechanisms of TQ-6, a Novel Ruthenium-Derivative Compound, against Lipopolysaccharide-Induced In Vitro Macrophage Activation and Liver Injury in Experimental Mice: The Crucial Role of p38 MAPK and NF-κB Signaling

**DOI:** 10.3390/cells7110217

**Published:** 2018-11-19

**Authors:** Chih-Hsuan Hsia, Marappan Velusamy, Thanasekaran Jayakumar, Yen-Jen Chen, Chih-Wei Hsia, Jie-Heng Tsai, Ruei-Dun Teng, Joen-Rong Sheu

**Affiliations:** 1 Graduate Institute of Medical Sciences, College of Medicine, Taipei Medical University, Taipei 110, Taiwan; d119102013@tmu.edu.tw (C.-H.H.); tjaya_2002@yahoo.co.in (T.J.); m120104004@tmu.edu.tw (Y.-J.C.); d119106003@tmu.edu.tw (C.-W.H.); tang0803@tmu.edu.tw (R.-D.T.); 2 Department of Chemistry, North Eastern Hill University, Shillong 793022, India; mvelusamy@gmail.com; 3 Department of Pharmacology, School of Medicine, College of Medicine, Taipei Medical University, Taipei 110, Taiwan; 4 School of Nutrition and Health Sciences, College of Nutrition, Taipei Medical University, Taipei 110, Taiwan; a19851102@hotmail.com

**Keywords:** RAW 264.7 cells, ruthenium compound, LPS, NF-κB, nuclear translocation, mice liver injury

## Abstract

Several studies have reported that metal complexes exhibit anti-inflammatory activities; however, the molecular mechanism is not well understood. In this study, we used a potent ruthenium (II)-derived compound, [Ru(η6-cymene)2-(1H-benzoimidazol-2-yl)-quinoline Cl]BF4 (TQ-6), to investigate the molecular mechanisms underlying the anti-inflammatory effects against lipopolysaccharide (LPS)-induced macrophage activation and liver injury in mice. Treating LPS-stimulated RAW 264.7 cells with TQ-6 suppressed nitric oxide (NO) production and inducible nitric oxide synthase (iNOS) expression in a concentration-dependent manner. The LPS-induced expression of tumor necrosis factor alpha (TNF-α) and interleukin-1 beta (IL-1β) were reduced in TQ-6-treated cells. TQ-6 suppressed, LPS-stimulated p38 MAPK phosphorylation, IκBα degradation, and p65 nuclear translocation in cells. Consistent with the in vitro studies, TQ-6 also suppressed the expression of iNOS, TNF-α, and p65 in the mouse model with acute liver injury induced by LPS. The present study showed that TQ-6 could protect against LPS-induced in vitro inflammation in macrophage and in vivo liver injury in mice, and suggested that NF-κB could be a promising target for protecting against LPS-induced inflammation and liver injury by TQ-6. Therefore, TQ-6 can be a potential therapeutic agent for treating inflammatory diseases.

## 1. Introduction

Inflammation is a complex process mediated by the activation of inflammatory and immune cells. During the inflammation process, macrophages play a central role in activating many different immunopathological phenomena, including the overproduction of pro-inflammatory cytokines and inflammatory mediators of interleukin (IL)-1β, tumor necrosis factor-α (TNF-α), and nitric oxide (NO) [1]. The model most commonly used to investigate induced inflammation is the stimulation of macrophages with lipopolysaccharide (LPS) [2]. LPS is an active pathogen-related molecular agent and a cell wall component of gram-negative bacteria. It is considered the most potent immunostimulant among all bacterial cell wall components [2]. Stimulating macrophages with LPS activates inflammatory signaling cascades via triggering toll-like receptor 4 (TLR4) complexes. The interaction recruits proteins, including myeloid differentiation primary response gene 88 and interleukin-1 receptor-associated kinases, and stimulates TNF receptor-associated factor 6 (TRAF6), which subsequently activates the nuclear factor (NF)-κB [3]. Subsequent to stimulation with LPS, NF-κB is activated by the inhibitor of the κB (IκB)-kinase (IKK) complex [4]. IKK phosphorylates IκB, causing IκB proteasomal degradation and the release of NF-κB [4]. The liberated dimeric NF-κB (p65 and p50 subunits) then translocates to the nucleus and activates the transcription of pro-inflammatory target genes that encode regulatory proteins. This leads to physiological responses, including inflammatory or immune responses [4].

The mitogen-activated protein kinase (MAPK) family, including extracellular signal-regulated kinase (ERK), c-Jun NH2-terminal kinase (JNK), and p38 MAPK, are the members of downstream targets of LPS-induced inflammatory cascades in macrophages [5]. When macrophages are stimulated with LPS, MAPKs are activated and produce inflammatory factors through the activation of multiple downstream signaling events [5]. LPS has also been identified as the most common agent to induce liver injury [6]. LPS-induced liver injury in mice has been employed as a model for molecular pathological research, mimicking the course of liver damage and septic endotoxemia or sometimes septic shock or death [6]. The mechanisms of acute liver injury are complex and not yet completely understood; however, it is generally accepted that inflammatory and oxidant injuries are involved [6]. Some studies have shown that NF-κB plays an essential role in the regulation of inflammatory signaling pathways in the liver [7]. Therefore, treatments for inhibiting NF-κB may have potential therapeutic advantages in alleviating inflammatory diseases. Despite the importance of inflammation in innate immunity, excess inflammatory responses are known to cause pathological conditions, such as rheumatoid arthritis and sepsis. Consequently, developing new therapeutic agents is essential for therapeutic control of inflammatory diseases [8].

Over the past few decades, various metal complexes have emerged as viable alternatives to organic molecules as beneficial agents [9]. The classical platinum drug cisplatin and its analogs, carboplatin and oxaliplatin, have been widely studied for their potent anticancer activity [10]. The copper-aspirin complex exhibited more potent anti-inflammatory effects than aspirin in rats [11]. Kale and co-workers found that zinc complex reduced inflammatory edema induced by carrageenan in the paws of rats [12]. Some interesting studies also reported that copper (II), cobalt (II), and manganese (II) complexes showed potent anti-inflammatory activities [9]. Our previous studies demonstrated the importance of ruthenium-based organometallic complexes to the development of effective antiplatelet agents for the prevention and treatment of thrombotic diseases [13]. In this study, we examined these anti-inflammatory effects and aimed to elucidate the possible mechanisms of TQ-6 in both LPS-stimulated RAW 264.7 cells and the mouse liver injury model.

## 2. Materials and Methods

### 2.1. Chemicals and Reagents

Fetal bovine serum (FBS), Dulbecco’s modified Eagle medium (DMEM), L-glutamine penicillin/streptomycin, and anti-α-tubulin monoclonal antibodies (mAbs) were purchased from Invitrogen (Thermo Fisher Scientific, Waltham, MA, USA). LPS (*Escherichia coli* 0127:B8), 3-(4,5-dimethylthiazol-2-yl)-2,5-diphenyltetrazolium bromide (MTT), dimethyl sulfoxide (DMSO), and 4-(4-fluorophenyl)-2-(4-methylsulfinylphenyl)-5-(4-pyridyl)-1H-imidazole (SB203580) were purchased from Sigma-Aldrich (St. Louis, MO, USA). Anti-Lamin B1 and anti-iNOS polycloncal antibody (pAb) were purchased from Santa Cruz Biotechnology (Dallas, TX, USA). The anti-TNF-α, anti-JNK, anti-phospho-c-JNK (Thr183/Tyr185), anti-phospho-p44/p42 ERK (Thr202/Tyr204), anti-phospho-p38 MAPK (Thr180/Tyr182) pAbs, and anti-phospho-p65 (Ser536), anti-p65, anti-IκBα, anti-ERK and anti-p38 MAPK mAbs were purchased from Cell Signaling (Danvers, MA, USA). Anti-IL-1β pAb was purchased from BioVision (Milpitas, CA, USA). Horseradish peroxidase (HRP)-conjugated donkey anti-rabbit immunoglobulin G (IgG), and sheep anti-mouse IgG were purchased from Amersham (Buckinghamshire, UK). The Western blotting detection reagent of enhanced chemiluminescence (ECL) and Hybond™-P polyvinylidene difluoride (PVDF) blotting membranes were purchased from GE Healthcare Life Sciences (Waukesha, WI, USA).

### 2.2. TQ-6 Synthesis and RAW 264.7 Cell Cultivation

The TQ-6 and its ligand (L) were synthesized according to the method described in our previous study [13]. RAW 264.7 cells were purchased from ATCC (ATCC number: TIB-71). The cells were cultured in DMEM supplemented with 10% FBS and 100 U/mL penicillin G and 100 mg/mL streptomycin at 37 °C in a humidified atmosphere of 5% CO_2_/95% air [14].

### 2.3. Cell Viability Assay

RAW 264.7 cells (2 × 10^5^ cells per well) were seeded into 24-well culture plates with DMEM containing 10% FBS for 24 h. The cells were treated with various concentrations of TQ-6 (5, 10 and 20 μM) or solvent control (0.1% DMSO) for 20 min, and then stimulated with LPS (1 μg/mL) or left unstimulated for 24 h. Cell viability was measured by using MTT assay [14]. The cell viability index was calculated as follows: (absorbance of treated-cells/absorbance of control cells) × 100%. The absorbance of samples was determined at 570 nm by an MRX absorbance reader (Dynex Technologies, Chantilly, VA, USA).

### 2.4. Determination of Nitric Oxide Production

To determine NO production, the level of nitrite/nitrate, stable oxidative end products of nitric oxide, was measured as previously described [14] with minor modifications. 8 × 10^5^ RAW 264.7 cells were seeded into 6-cm dishes with DMEM containing 10% FBS for 24 h. The cells were treated with TQ-6 (5–20 μM) or solvent control (0.1% DMSO) for 20 min and then stimulated with LPS (1 μg/mL) or left unstimulated for 24 h. These conditioned supernatants were collected and mixed with equal volumes of Griess reagent (1% sulphanilamide and 0.1% naphthalenediamine dissolved in 2.5% phosphoric acid). The absorbance of samples was determined at 550 nm by an MRX absorbance reader. The concentrations of nitrite/nitrate were calculated by a standard curve performed through the linear regression of absorbance measurements of standard solutions (sodium nitrite dissolved in the same culture medium).

### 2.5. Separation of Cytoplasmic and Nuclear Extracts

RAW 264.7 cells (8 × 10^5^ cells per dish) were treated with 0.1% DMSO or 20 μM TQ-6 with or without LPS stimulation for 30 min in 6-cm dishes and were maintained in a humidified atmosphere. Subsequently, the cells were harvested, and cytoplasmic and nuclear proteins were extracted using the NE-PER kit (Thermo Fisher Scientific, Waltham, MA, USA) according to the manufacturer’s instructions. Lamin B1 and α-tubulin were used as internal controls for the nucleus and cytosol, respectively [15].

### 2.6. Immunofluorescence Staining Assay

RAW 264.7 cells (5 × 10^4^ cells per well) were cultured on cover slips in 6-well plates and treated with 0.1% DMSO or 20 μM TQ-6 with or without LPS stimulation for 30 min. The cells were washed with phosphate-buffered saline (PBS) and fixed with 4% paraformaldehyde in PBS for 10 min at room temperature. After incubation, the cells were permeabilized with 0.1% Triton X-100 for 10 min and blocked with 5% BSA for 30 min. The cells were incubated with primary antibodies overnight at 4 °C, subsequently washed 3 times with PBS, and incubated with secondary antibodies for 1 h at room temperature. The samples were stained with 4′,6-diamidino-2-phenylindole (DAPI, 30 μM) and mounted using a mounting buffer (Vector Laboratories) on a glass slice. The samples were detected under a Leica TCS SP5 confocal spectral microscope imaging system using an argon or krypton laser (Mannheim, Germany) [15].

### 2.7. LPS-Induced Acute Liver Inflammation in Mice

Male (eight-week-old) C57BL/6 mice were used for this study. All animal experiments and care procedures conformed to the Guide for the Care and Use of Laboratory Animals (LAC-2016-0395) and were approved by the Institutional Animal Care and Use Committee of Taipei Medical University. The mice were divided into four groups: control, LPS (2.5 mg/kg), TQ-6 (1 mg/kg) + LPS (2.5 mg/kg), and TQ-6 (2 mg/kg) + LPS (2.5 mg/kg). The mice were initially pretreated intraperitoneally (i.p.) with TQ-6 or 0.1% DMSO. After the administration of TQ-6 for 2 h, the LPS was then injected with i.p. The mice were sacrificed after 2 or 6 h of LPS stimulation, and liver tissues were quickly removed and stored at −80 °C until analysis.

### 2.8. Western Blotting

A Western blotting analysis was performed to determine the protein expression in cells and tissue homogenates, as previously described [14,15]. RAW 264.7 cells (8 × 10^5^ cells/dish) were seeded on 6-cm dishes with DMEM containing 10% FBS for 24 h. The cells were pretreated with TQ-6 or 0.1% DMSO for 20 min and either stimulated with LPS (1 μg/mL) or left unstimulated, according to the experimental design. Subsequently, the proteins from the cells and liver tissues were extracted using lysis buffer. The extracted protein samples (50 μg) were applied to sodium dodecyl sulphate (SDS)-polyacrylamide gel electrophoresis, and the separated proteins were then electrophoretically transferred onto PVDF membranes (0.45 μm). The membranes were blocked with 5% skimmed milk in TBST buffer (10 mM Tris-base, 100 mM NaCl and 0.01% Tween 20) for 30 min. The membranes were incubated with the targeting primary antibodies against iNOS, TNF-α, IL-1β, phospho-p38 MAPK, phospho-c-JNK, phospho-p44/p42 ERK, IκBα, and phospho-p65 for 2 h, and then subjected to HRP-conjugated donkey anti-rabbit IgG or sheep anti-mouse IgG for 1 h at room temperature. The ECL system was used to detect the immune-reactive bands. The densitometry of protein bands was performed by the Biolight Windows Application, V2000.01 (Bio-Profil, Vilber Lourmat, France).

### 2.9. Statistical Analysis

The results are presented as the means ± standard error (SEM) and are accompanied by the number of observations (*n*). Data were assessed using one-way analysis of variance (one-way ANOVA). If one-way ANOVA revealed significant differences among the group means, a subsequent comparison of Newman-Keuls method was performed. A *p* value < 0.05 was considered as a statistically significant difference.

## 3. Results

### 3.1. Effects of TQ-6 on Cytotoxicity and Morphology in RAW 264.7 Cells

**Figure 1:**
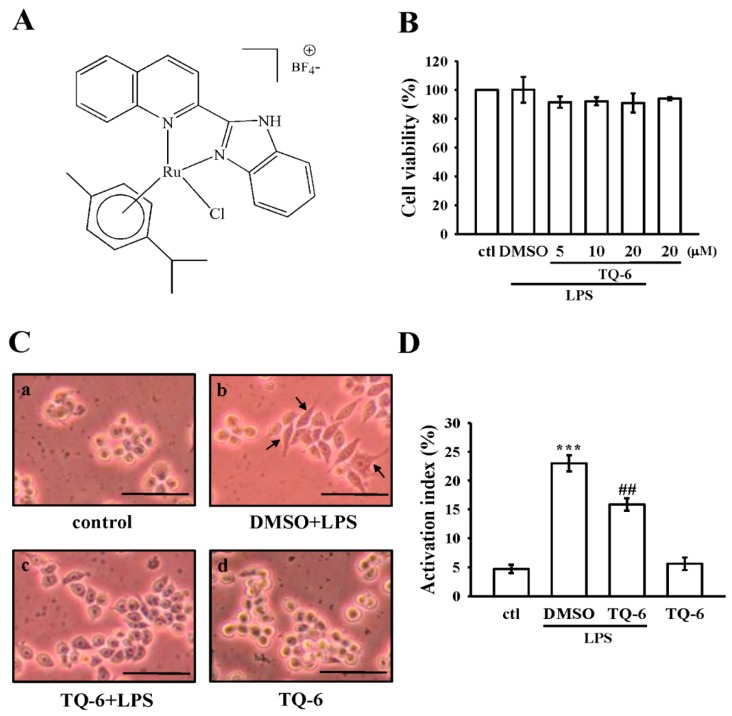
Effects of [Ru(η6-cymene)2-(1H-benzoimidazol-2-yl)-quinoline Cl]BF4 (TQ-6) on cell viability and morphological changes in lipopolysaccharide (LPS)-stimulated RAW 264.7 cells. (**A**) Chemical structure of TQ-6. (**B**) Cells were treated with 0.1% DMSO or pre-treated with TQ-6 (5–20 μM) for 20 min and then treated with LPS (1 μg/mL) for 24 h. Cell viability was evaluated as described in the Methods section. (**C**) Cell morphology was observed using optical microscopy. Black bar = 50 μm. Activated cells are indicated by arrows. (**D**) The activation index percentage was shown as the number of cells with activated morphology relative to the total number of cells. Data are presented as the means ± SEM (*n* = 4). *** *p* < 0.001, compared with the control group; ## *p* < 0.01, compared with the LPS group.

The cytotoxicity of TQ-6 (Figure 1A) on RAW 264.7 cells was first examined using the MTT assay. TQ-6 treatment (5, 10, and 20 μM) did not exert significant cytotoxic effects in LPS-stimulated RAW cells (Figure 1B). In addition, microscopical cell morphology showed that unstimulated RAW cells had a round morphology (Figure 1(Ca)), whereas LPS-stimulated cells had an irregular morphology, as well as pseudopodia formation and cell spreading (Figure 1(Cb)). This alteration was diminished by TQ-6 pretreatment (Figure 1(Cc)). Moreover, TQ-6 (20 μM) did not significantly affect the round morphology in unstimulated cells (Figure 1(Cd)). As shown in Figure 1D, quantitative analysis revealed that LPS significantly increased the number of cells with dendritic morphology (23.0 ± 1.4% as compared to the control 4.7 ± 0.7%; *p* < 0.001, *n* = 4). This increment was significantly decreased when cells were pretreated with 20 μM TQ-6 (15.9 ± 1.1% as compared to LPS treatment; *p* < 0.01, *n* = 4).

### 3.2. Effect of TQ-6 on NO Production and iNOS Expression in RAW Cells

**Figure 2:**
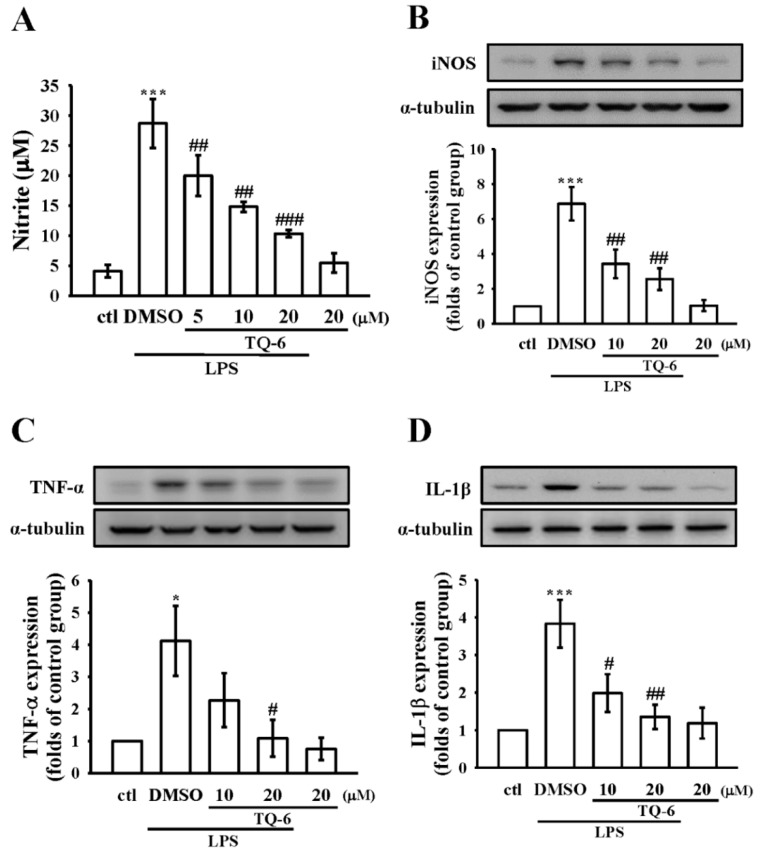
Effects of TQ-6 on nitric oxide (NO) production, nitric oxide synthase (iNOS) expression, tumor necrosis factor alpha (TNF-α), and interleukin-1 beta (IL-1β) production in LPS-stimulated RAW cells. (**A**) Cells were pretreated with TQ-6 (5, 10, and 20 μM) for 20 min and then stimulated by LPS (1 μg/mL) for 24 h. NO was measured using Griess reagent; (**B**–**D**) Cells were pretreated with TQ-6 (10 and 20 μM) for 20 min and then stimulated by LPS (1 μg/mL) for 24 h. The levels of (**B**) iNOS, (**C**) TNF-α, and (**D**) IL-1β protein expression were evaluated as described in the Methods section. Data are presented as the means ± SEM (*n* = 4); * *p* < 0.05 and *** *p* < 0.001, compared with the control group; # *p* <0.05, ## *p* < 0.01, and ### *p* < 0.001, compared with the LPS group.

As shown in Figure 2A, treatment with TQ-6 significantly inhibited the LPS-induced NO production in RAW cells in a concentration-dependent manner. More precisely, compared with control cells, LPS treatment markedly increased NO production (4.1 ± 1.1 μM versus 28.7 ± 4.1 μM, *n* = 4), and co-treatment with TQ-6 at 5, 10, and 20 μM significantly decreased its production to about 20 ± 3.4, 14.8 ± 0.8, and 10.3 ± 0.6 μM, respectively. We next determined whether the expression of the iNOS protein, which catalyzes NO generation, was inhibited by TQ-6 in LPS-activated cells. Compared with the control cells, LPS treatment significantly increased iNOS expression, whereas this expression inhibited concentration dependency by TQ-6 (*p* < 0.01, *n* = 4; Figure 2B). These results suggested that the TQ-6-mediated inhibition of NO formation was a consequence of the down-regulation of iNOS expression in LPS-stimulated cells.

### 3.3. Effect of TQ-6 on Pro-Inflammatory Cytokines Expression in RAW Cells

The release of pro-inflammatory cytokines, such as TNF-α and IL-1β, is one of the most important features of the LPS-induced inflammatory response. Therefore, to clarify whether TQ-6 affected cytokine production in RAW cells, a Western blot assay was conducted. The results are shown in Figure 2C,D, and they indicate that when RAW cells are treated with TQ-6 alone, there are no significant changes over the production of TNF-α or IL-1β, as they are comparable with the control cells. However, the production of these cytokines were increased in LPS-stimulated cells, and these elevations were significantly reduced by TQ-6.

### 3.4. The Influence of TQ-6 on MAPKs in LPS-Induced RAW Cell Activation

**Figure 3:**
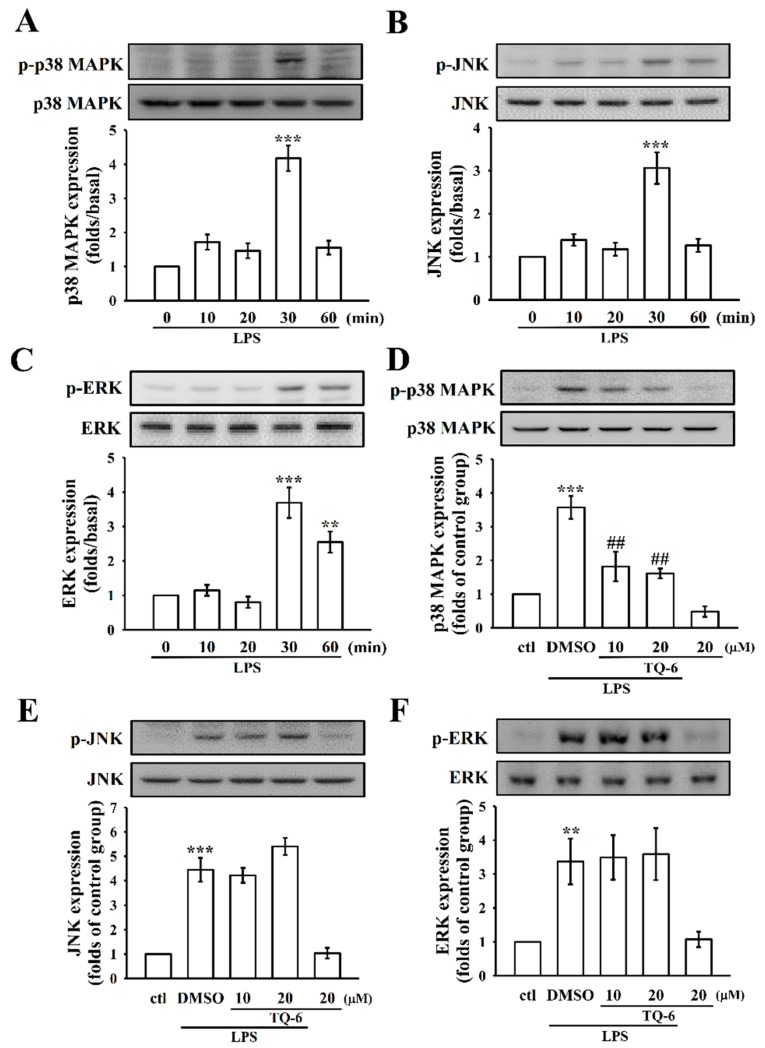
The influence of TQ-6 on LPS-induced phosphorylation of p38 mitogen-activated protein kinase (MAPK), c-Jun NH2-terminal kinase (JNK), and extracellular signal-regulated kinase (ERK) in RAW cells. (**A**–**C**) Cells were treated with LPS (1 μg/mL) for the indicated times (10–60 min) and the phosphorylation of (**A**) p38 MAPK, (**B**) JNK, and (**C**) ERK were determined by immunoblotting as described in the Methods. (**D**–**F**) Cells were treated with 0.1% DMSO or TQ-6 (10 and 20 μM) for 20 min, followed by LPS (1 μg/mL) for 30 min, and the phosphorylation of (**D**) p38 MAPK, (**E**) JNK, and (**F**) ERK were evaluated by immunoblotting. Data are presented as the means ± SEM (*n* = 4). ** *p* < 0.01 and *** *p* < 0.001, compared with the control group; ## *p* < 0.01, compared with the LPS group.

Activation of MAPKs is known to be involved with LPS-induced, pro-inflammatory cytokines [5]. To investigate whether TQ-6 regulated the activation of MAPKs in response to LPS, the level of phosphorylated p38 MAPK, JNK, and ERK were determined by different time courses of LPS-stimulated RAW cells. The results showed that the expressions of all three MAPKs phosphorylation peaked at 30 min after LPS treatment (Figure 3A–C). As shown in Figure 3D–F, LPS significantly increased the phosphorylation of p38 MAPK, JNK, and ERK in RAW cells, and pretreatment with 10 or 20 μM TQ-6 was only effective at inhibiting the elevated phosphorylation of p38 MAPK (Figure 3D); however, the JNK and ERK did not alter by TQ-6 (Figure 3E,F).

### 3.5. TQ-6 Regulates LPS-Induced NF-κB Activation in RAW Cells

**Figure 4:**
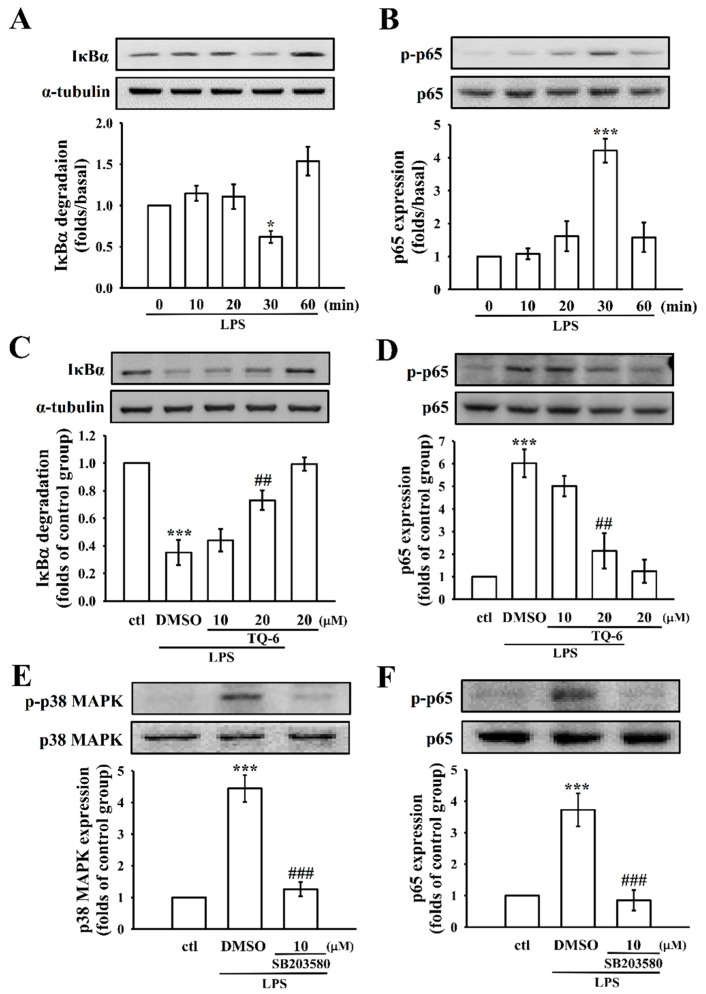
Time-course analysis and the effects of TQ-6 on LPS-induced IκBα degradation and p65 phosphorylation in RAW cells. (**A**,**B**) Cells were treated with LPS (1 μg/mL) for the indicated times (10–60 min). (**A**) IκBα degradation and (**B**) p65 phosphorylation were determined by immunoblotting, as described in the Methods section. (**C**–**F**) Cells were treated with 0.1% DMSO, TQ-6 (10 and 20 μM), or SB203580 (p38 MAPK inhibitor,10 μM) for 20 min, followed by LPS (1 μg/mL) for 30 min. (**C**) IκBα degradation, (**D**,**F**) phosphorylation of p65, and (**E**) phosphorylation of p38 MAPK were determined by immunoblotting. Data are presented as the means ± SEM (*n* = 4). * *p* < 0.05 and *** *p* < 0.001, compared with the control group; ## *p* < 0.01 and ### *p* < 0.001 compared with the LPS group.

The expressions of pro-inflammatory cytokines and proteins, such as TNF-α, IL-1β, and iNOS, are directly associated with the IκBα-NF-κB signaling cascades [4]. A time-course analysis revealed that IκBα degradation and p65 phosphorylation peaked at 30 min in LPS-treated cells, as shown in Figure 4A,B; therefore, this time-point was selected for the subsequent experiments. TQ-6 (20 μM) treatment reversed LPS-stimulated IκBα degradation and p65 phosphorylation (Figure 4C,D). This result indicates that NF-κB signaling may play an important role on TQ-6’s inhibitory effects on LPS-stimulated, pro-inflammatory cytokine expressions.

In addition, we performed another study to establish whether the p38 MAPK acted as an upstream regulator of p65 in LPS-induced RAW cells, where the cells were exposed to LPS with or without 10 μM of SB203580 (an inhibitor of p38 MAPK). This hypothesis was confirmed by the noted result that SB203580 significantly inhibited the LPS-induced phosphorylation of p65 in RAW cells (Figure 4F).

### 3.6. TQ-6 Attenuated, LPS-Induced NF-κB Nuclear Translocation in RAW Cells

**Figure 5:**
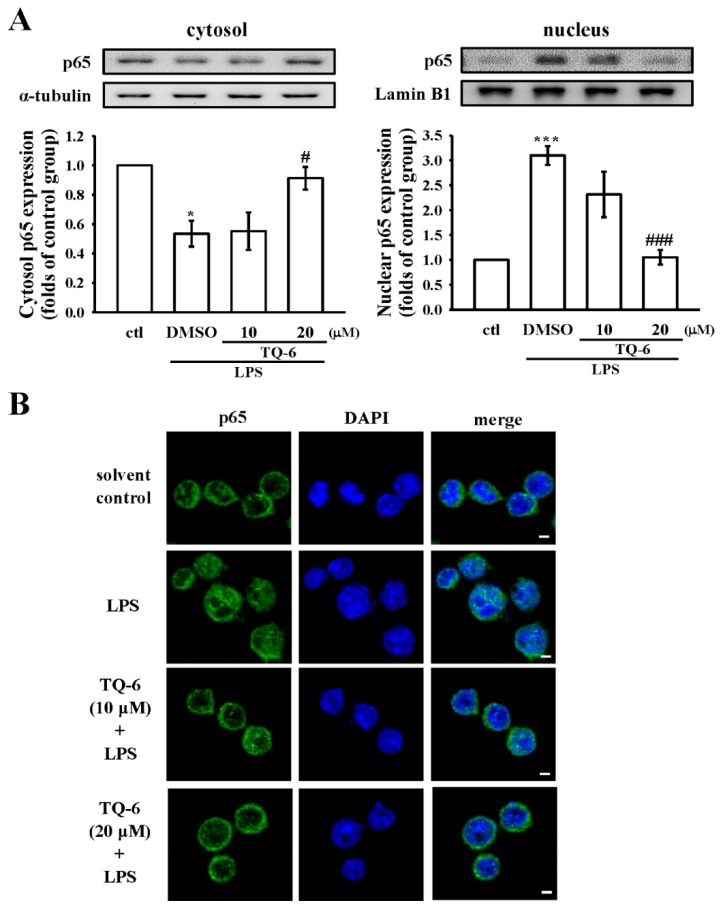
Effects of TQ-6 on LPS-induced nuclear translocation of NF-κB subunit p65. RAW cells were treated with TQ-6 (10 and 20 μM) and LPS (1 μg/mL) for 30 min. (**A**) The cytosolic and nuclear fractions were isolated using the NE-PER kit and then subjected to Western blotting to detect p65 expression. Lamin B1 and α-tubulin were used as internal controls for the nucleus and cytosol, respectively. (**B**) The immunofluorescence analysis was performed with an anti-p65 antibody and FITC-conjugated anti-rabbit IgG antibody (green). 4′,6-diamidino-2-phenylindole (DAPI) was used to label the nuclei (blue). The images were captured by confocal microscopy (scale bar = 2.5 μm). Data are presented as the means ± SEM (*n* = 4). * *p* < 0.05 and *** *p* < 0.001, compared with the control group; # *p* < 0.05 and ### *p* < 0.001, compared with the LPS group.

Following inflammatory stimulation, NF-κB is activated by post-translational modifications, and translocates into the nucleus to induce the transcription of pro-inflammatory genes [4]. To determine the effect of TQ-6 on the nuclear translocation of the NF-κB p65 subunit, the RAW cells were treated with TQ-6 and then stimulated with LPS. As shown in Figure 5A, the level of total p65 proteins was markedly decreased in cytosolic fractions, which was associated with the increase in the nuclear fraction of the LPS-stimulated cells, whereas TQ-6 (20 μM) treatment significantly reversed the p65 accumulation in both cytosolic and nuclear fractions.

Next, an immunofluorescence assay was carried out to confirm the inhibition of LPS-induced p65 nuclear translocation by TQ-6. LPS treatment induced the translocation of p65 from the cytoplasm to the nucleus, and TQ-6 prevented this translocation (Figure 5B). Compared to control cells, LPS treatment markedly increased p65 nuclear export, as evidenced by the amplified FITC labeled NF-κB p65 (green fluorescence) in the RAW cell nuclei. However, treatment with TQ-6 at 20 μM for 30 min blocked the nuclear translocation of p65, which was confirmed by noticeably reduced green fluorescence in the nuclear fraction.

### 3.7. TQ-6 Restores Inflammation in LPS-Induced Liver Injury

**Figure 6:**
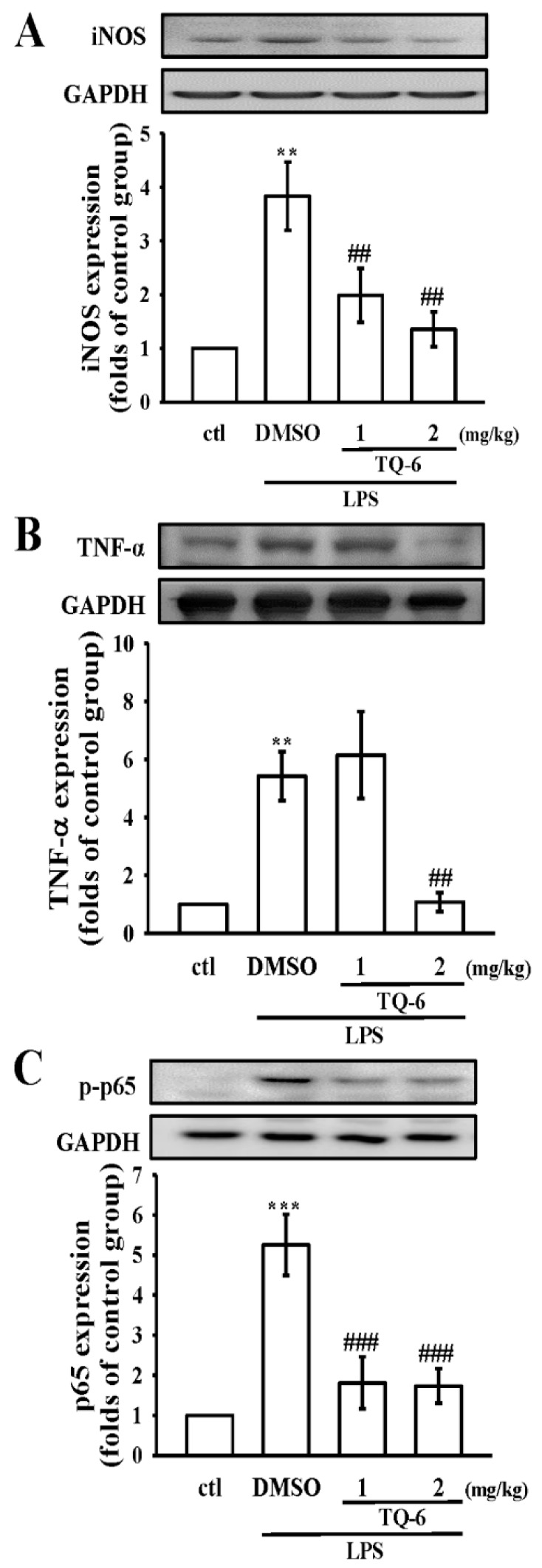
Effects of TQ-6 on the expression of iNOS, TNF-α, and p65 proteins in liver tissues of LPS-induced mice. Mice were given an intraperitoneal injection of TQ-6 (1 and 2 mg/kg) 2 h prior to administration of LPS (2.5 mg/kg). After a 6 h LPS stimulation, the protein expression of (**A**) iNOS, (**B**) TNF-α, and (**C**) p65 were analyzed. Data presented are the means ± SEM (*n* = 6); ** *p* < 0.01 and *** *p* < 0.001, compared with the control group; ## *p* < 0.01 and ### *p* < 0.001, compared with the LPS group.

Liver cells play a major role in immunological homeostasis and metabolism, while LPS, inflammatory factors, and pathogens impair these crucial functions [16]. Large amounts of NO, derived from the high-capacity iNOS, are generated during endotoxemia [17]. In this study, iNOS expression was potently induced in LPS-exposed liver tissues (Figure 6A), while pretreatment with TQ-6 (1 and 2 mg/kg) blunted this effect. Similarly, the data showed that LPS enhanced the protein expression of TNF-α and upregulated the phosphorylation of p65. Contrarily, TQ-6 diminished the expressions of these proteins in LPS-injured livers of mice (Figure 6B,C). Together, the results of the in vitro experiments and their consistent in vivo findings propose that the NF-κB signaling pathway may evidently participate in the anti-inflammatory effect of TQ-6 in the LPS-induced RAW cells and the mice model.

## 4. Discussion

Metal complexes possess several notable advantages that render them as attractive alternatives to organic small molecules for the development of therapeutic agents [9]. However, relatively less attention has been devoted to the development of metal complexes for anti-inflammatory applications. Notably, in this study, it has been demonstrated that a novel ruthenium compound, TQ-6, exhibits effective anti-inflammatory properties via inhibiting inflammatory mediators (NO and iNOS) and pro-inflammatory cytokines (TNF-α and IL-1β), and blocking LPS-induced p38 MAPK/p65 phosphorylation, IκBα degradation, and p65 nuclear translocation in RAW 264.7 cells. Additionally, TQ-6 protects against LPS-induced liver injury in mice through blocking p65 activation and subsequently suppressing TNF-α and iNOS expression. These data demonstrated that TQ-6 exhibited potent anti-inflammatory activity via mediating the inhibition of NF-κB-signaling pathways.

NO is produced through the action of iNOS, and participates in diverse biological mechanisms as a potent pro-inflammatory mediator [18]. Numerous studies have revealed that excessive NO production is vital to the pathogenesis of inflammation, and can also lead to tissue damage [17]. iNOS is present at a low level under normal physiological conditions; however, it gets induced rapidly by LPS [17]. Several inhibitors of NO have been reported to exert their anti-inflammatory effects by preventing iNOS expression [19]. Liu et al. reported that the rhodium (III) complex inhibits LPS-induced NO production in RAW 264.7 cells [20]. Consistent with this finding, in our present study, TQ-6 inhibited NO production by hindering iNOS expression to demonstrate its anti-inflammatory response.

Macrophages play important roles in the host defense to infection, repair of damaged tissue, and secretion of pro-inflammatory cytokines to modulate inflammatory responses [21]. TNF-α and IL-1β are majorly secreted cytokines that mediate and regulate inflammatory diseases [22]. TNF-α plays a major role in the cascade of pro-inflammatory cytokines and the subsequent inflammatory processes [23]. IL-1β is implicated in the pathophysiology changes that occur during different disease states, such as rheumatoid arthritis, inflammatory bowel disease, vascular disease, and so forth [22]. The overwhelming secretion of these cytokines is reported to cause severe tissue damage, multiple organ failure, or even death [24]. Therefore, repressing the overproduction of pro-inflammatory cytokines would be a therapeutic strategy for controlling inflammatory diseases. Consistent with this finding, a study indicated that gold (I) complexes significantly reduced the production of TNF-α and IL-1β in LPS-activated macrophages [9]. In addition, Zhong et al. [25] also found that rhodium (III) complex inhibited the release of TNF-α and IL-1β induced by LPS in RAW264.7 cells. In this study, TQ-6 significantly suppressed TNF-α and IL-1β production. These findings suggest that TQ-6 may inhibit pro-inflammatory cytokines to attenuate inflammatory responses.

The release of inflammatory mediators during inflammatory diseases is controlled by the activation of intracellular signaling cascades. The TLRs signal pathway is activated by the binding of LPS, which triggers the downstream of MAPKs and NF-κB pathways [4,5]. Three families of MAPKs (ERK, JNK, and p38 MAPK) play critical roles in cell growth regulation and differentiation, in addition to the control of cellular responses to cytokines and stressors [26]. Moreover, it has been recognized that MAPKs play a pivotal role in the pathogenesis of many inflammatory disorders [27]. A study demonstrated that manganese (II) complex diminishes the gene expression and protein secretion of cytokines induced by LPS in THP-1 macrophages, probably due in part to the inactivation of the MAPK signaling pathway [28]. Recent in vitro studies in RAW 264.7 cells have shown that p38 MAPK is involved in NF-κB activation [29], and our data also proved this hypothesis in that the p38 inhibitor, SB203508, inhibited p65. Moreover, TQ-6 exclusively regulated p38 MAPK phosphorylation in LPS-stimulated RAW 264.7 cells. This result supports the finding that TQ-6 affects pro-inflammatory signaling through regulating NF-κB activation via p38 MAPK.

MAPK phosphorylation activates the transcription of NF-κB-mediated pro-inflammatory cytokines [4]. The NF-κB family consists of five proteins—p65 (RelA), RelB, c-Rel, p105/p50 (NF-κB1), and p100/52 (NF-κB2)—which associate with each other to form diverse, transcriptionally-active homo and heterodimeric complexes. From the canonical and non-canonical signaling of NF-κB, only the canonical pathway (p65/p50 dimers) is induced by TNFα, IL-1, or LPS to engage IKK activity [30]. In LPS-induced inflammation, NF-κB is released and transferred from the cytoplasm to the nucleus, which leads to the overexpression of several inflammatory mediators, including iNOS, TNF-α, IL-1β, and IL-6 [4]. In Jurkat T cells, Cu^2+^ was shown to inhibit NF-κB activation through blocking signal-induced degradation of IkBα [31]. Another study demonstrated that zinc and copper complexes inhibited NF-κB activation in LPS-stimulated RAW 264.7 cells [32]. Comparable with these results, TQ-6 was shown to inhibit IκBα degradation, NF-κB phosphorylation, and nuclear translocation. Altogether, our results suggested that TQ-6-mediated inhibition of LPS-induced pro-inflammatory cytokine production may be possible via the regulation of NF-κB signaling cascades.

The importance of macrophage activation and LPS-mediated induction of pro-inflammatory factors in liver injury is evident from numerous models of liver disorders. A previous study showed that LPS triggered liver injury by elevating TNF-α in a non-alcoholic steatohepatitis (NASH) model [33]. In a mouse model of intestinal injury, LPS was found to mediate liver injury by increasing TNF-α and IL-6 through TLR activation [34]. In a model of ischemia-reperfusion liver injury, LPS also promoted the induction of pro-inflammatory cytokines, such as TNF-α, IL-6, and IL-1β [35]. Interfering with the LPS-induced inflammatory response would be beneficial to cope with inflammation-associated liver disorders. In the present study, TQ-6 was shown to suppress NF-κB, TNF-α, and iNOS expression in LPS-challenged mice. Collectively, these findings suggest that TQ-6 inhibits LPS-induced liver damage via suppressing NF-κB signaling.

This study examined the possible mechanism of anti-inflammatory action of a newly synthesized ruthenium compound, TQ-6, using in vitro RAW 264.7 cells and in vivo mice liver injury models. Our results demonstrated that TQ-6 attenuates the LPS-induced activation of p38 MAPK and NF-κB, contributing to the anti-inflammatory effects by reducing the levels of TNF-α and IL-1β and inhibiting the expression of iNOS. The inhibition of LPS-induced p65 nuclear translocation by TQ-6, and the reliable findings of the in vivo mice model may support the outcome of this study. Therefore, this study suggests that TQ-6 may be considered as a potential candidate for the treatment of inflammation-related diseases.
